# Detection of Middle East respiratory syndrome coronavirus using reverse transcription loop-mediated isothermal amplification (RT-LAMP)

**DOI:** 10.1186/1743-422X-11-139

**Published:** 2014-08-08

**Authors:** Kazuya Shirato, Takuya Yano, Syouhei Senba, Shigehiro Akachi, Takashi Kobayashi, Takamichi Nishinaka, Tsugunori Notomi, Shutoku Matsuyama

**Affiliations:** 1 Laboratory of Acute Respiratory Viral Diseases and Cytokines, Department of Virology III, National Institute of Infectious Disease, Laboratory of Acute Respiratory Viral Diseases and Cytokines, 4-7-1 Gakuen, Musashimurayama, Tokyo 208-0011, Japan; 2 Mie Prefecture Health and Environment Research Institute, 3684-11 Sakura-cho, Yokkaichi, Mie 512-1211, Japan; 3 Eiken Chemical Co. Ltd., 4-19-9 Taito, Taito-ku, Tokyo 110-8408, Japan

**Keywords:** Meddle East respiratory syndrome (MERS), MERS coronavirus (MERS-CoV), RT-LAMP, Genetic diagnostic method

## Abstract

**Background:**

The first documented case of Middle East Respiratory Syndrome coronavirus (MERS-CoV) occurred in 2012, and outbreaks have continued ever since, mainly in Saudi Arabia. MERS-CoV is primarily diagnosed using a real-time RT-PCR assay, with at least two different genomic targets required for a positive diagnosis according to the case definition of The World Health Organization (WHO) as of 3 July 2013. Therefore, it is urgently necessary to develop as many specific genetic diagnostic methods as possible to allow stable diagnosis of MERS-CoV infections.

**Methods:**

Reverse transcription-loop-mediated isothermal amplification (RT-LAMP) is a genetic diagnostic method used widely for the detection of viral pathogens, which requires only a single temperature for amplification, and can be completed in less than 1 h. This study developed a novel RT-LAMP assay for detecting MERS-CoV using primer sets targeting a conserved nucleocapsid protein region.

**Results:**

The RT-LAMP assay was capable of detecting as few as 3.4 copies of MERS-CoV RNA, and was highly specific, with no cross-reaction to other respiratory viruses. Pilot experiments to detect MERS-CoV from medium containing pharyngeal swabs inoculated with pre-titrated viruses were also performed. The RT-LAMP assay exhibited sensitivity similar to that of MERS-CoV real-time RT-PCR.

**Conclusions:**

These results suggest that the RT-LAMP assay described here is a useful tool for the diagnosis and epidemiologic surveillance of human MERS-CoV infections.

## Background

On 22 September 2012, a novel coronavirus sequence was detected from a 49-year-old patient presenting with severe pneumonia who was initially treated in an intensive care unit in Qatar and then moved to London [1]. The sequence of the PCR amplicon of this isolate was a close match with that of a coronavirus isolated from a 60-year-old patient who had died of severe pneumonia in Jeddah, Saudi Arabia in June 2012 [1,2]. Together, these two cases marked the beginning of an outbreak of severe respiratory infections caused by a newly identified coronavirus, designated the Middle East Respiratory Syndrome coronavirus (MERS-CoV) [3]. This outbreak is ongoing, with 836 confirmed cases to date that have resulted in 288 deaths in 19 countries (Jordan, Qatar, Saudi Arabia, the United Arab Emirates, Oman, Kuwait, Yemen, Lebanon, Iran, Algeria, Tunisia, France, the Netherlands, Germany, the United Kingdom, Greece, Malaysia, Philippines and the United States of America) as of 14 July, 2014 [The World Health Organization (WHO), Global Alert and Response (GAR), Coronavirus infections, updated on 14 July 2014, http://www.who.int/csr/disease/coronavirus_infections/en/index.html].

Sequence analyses show that MERS-CoV clusters with the group 2c betacoronavirus, and is closely related to the bat coronaviruses HKU4 and HKU5 [4]. Severe acute respiratory syndrome coronavirus (SARS-CoV), which caused severe pneumonia resulting in 8,098 reported infections and 774 deaths between 2002 and 2003 [5], was also derived from bat coronaviruses [6,7]. MERS-CoV, with its similar symptoms and phylogeny, is therefore considered a cousin of SARS-CoV. The reservoir for MERS-CoV remains unclear, but recent reports suggest that camels are the most likely candidate, as a form of the virus has been circulating in camels in Saudi Arabia since at least 1992 [8-13].

MERS-CoV is primarily diagnosed using a real-time RT-PCR assay, and at least two different genomic targets are required for a positive diagnosis. The first probe and primer sets for MERS-CoV detection by real-time PT-PCR were developed by Corman *et al*. shortly following the first reports of the disease [14,15]. Among them, the probe and primer sets targeting upE and ORF1a exhibit the highest sensitivities, and remain the most widely used targets for MERS-CoV detection. At least two different specific genomic targets are required for a positive diagnosis according to the case definition announced by the WHO as of 3 July 2013 [WHO, GAR, Revised interim case definition for reporting to WHO – Middle East respiratory syndrome coronavirus (MERS-CoV), updated on 3 July 2013, http://www.who.int/csr/disease/coronavirus_infections/case_definition/en/index.html]. A single positive target followed by gene sequencing is also considered positive; however, the current gene sequencing technique requires PCR amplicons, and the ability of conventional RT-PCR to produce a sequencing-quality template is generally lower than that of real-time RT-PCR [16-20]. Therefore, it is urgently necessary to develop as many specific genetic diagnostic methods as possible to allow reliable diagnosis of MERS-CoV infections.

The loop-mediated isothermal amplification (LAMP) method amplifies specific nucleic acid sequences using a set of four or six unique primers [21,22]. The LAMP procedure is user-friendly, since the reaction mixture is incubated at a single temperature for less than 1 h. Amplification can be detected as the precipitation of magnesium pyrophosphate or by fluorescence under ultra-violet light, and also can be detected in real time by monitoring the turbidity of the pyrophosphate [23]. The LAMP assay can also be used for the detection of RNA by combining reverse transcription with LAMP (RT-LAMP) [21]; RT-LAMP assays have been developed for a variety of respiratory RNA viruses, including SARS [24], respiratory syncytial virus [25,26], and influenza viruses [27,28]. In this study, a novel RT-LAMP method for the detection of MERS-CoV was developed, with a sensitivity similar to that of real-time RT-PCR targeting upE and ORF1a.

## Materials and methods

### Viruses

The MERS-CoV EMC isolate was kindly provided by Ron A. M. Fouchier, Erasmus Medical Center, Rotterdam, The Netherlands. MERS-CoV was propagated and titrated using Vero cells. Human respiratory syncytial viruses (RSV; Long, A2, B WV/14617/85 and 18537) were obtained from the American Tissue Culture Collection (ATCC). Human metapneumovirus (HMPV; Sendai-H/2404/2003) was obtained from the Virus Research Center, Sendai Medical Center, Japan. Human coronavirus (HCoV)-229E isolates ATCC VR-740 and Sendai-H/1121/04 [29] were used. HCoV-NL63 was supplied by Dr. Hoek, University of Amsterdam, Netherlands. Isolate HCoV-OC43 was obtained from ATCC. SARS coronavirus (Frankfurt strain) was supplied by Dr. J. Ziebuhr, University of Würzburg, Germany. Human parainfluenza viruses (PIV) 1 (strain C35) and 3 (strain C243) were obtained from ATCC. Adenoviruses (ADV) (serotype 3, strain G.B.; serotype 4, strain RI-67; and serotype 7, strain Gomen) were obtained from ATCC. Viruses were propagated and titrated using HEp-2, HeLa, RD, Vero cells, or LLC-Mk2 cells [30]. Influenza viruses [Flu; A/California/7/2009 (H1N1pdm), A/Victoria/210/2009 (H3N2), and B/Brisbane/60/2008] were provided by the Influenza Virus Research Center of the National Institute of Infectious Diseases in Japan, and were propagated and titrated using MDCK cells.

### Design of primer sets for RT-LAMP

Primer sets for the RT-LAMP assay were designed using the online LAMP primer design software (PrimerExplorer V4; http://primerexplorer.jp/e/) based on the nucleocapsid protein sequence of the EMC isolate of MERS-CoV (GenBank JX869059.2).

### Extraction of nucleic acids

RNA was extracted from viral stocks using TRIzol LS or TRIzol reagent (Invitrogen), according to the manufacturer’s instructions. Viral DNA was extracted using Qiagen Genomic-tip (Qiagen, Hilden, Germany), according to the manufacturer’s instructions. Total RNA and genomic DNA were quantitated using standard methods to measure the OD value. The MERS-CoV RNA copy number was calculated based upon the standard curve obtained using a TaqMan assay, as described by Corman *et al*. [14] (upE probe set and the positive control template). Total RNA was then diluted with ribonuclease-free water containing 10 μg/mL of Ribonucleic Acid from Baker’s Yeast (R6750; Sigma-Aldrich, St. Louis, MO, USA) as carrier RNA.

### RT-LAMP assay

The RT-LAMP assay was performed using the Loopamp RNA Amplification Kit (RT-LAMP; Eiken, Tokyo, Japan) under the following conditions: 5-μL sample (RNA or DNA) was mixed with 40 pmol each of FIP and BIP primers, 20 pmol each of LF and LB primers, 5 pmol each of F3 and B3 primers, 1-μL Enzyme Mix, and 12.5-μL Reaction Mix; distilled water was added to obtain a final volume of 25 μL. For real-time monitoring of RT-LAMP amplification, the reaction mixture was incubated at 65°C for 30 min in a Loopamp real-time turbidimeter (LA-320C, Eiken). For fluorescence detection, 1-μL Fluorescent Detection Reagent (Eiken) was added to the reaction mixture described above before the start of amplification, and then, fluorescence was detected under ultraviolet light after 30 min of amplification. Negative controls containing only yeast RNA were included in each assay.

To synthesize control RNA for RT-LAMP, the nucleocapsid protein sequence of the EMC strain (28566–29807) was amplified and cloned into the pGEM-T easy vector (Promega, Fitchburg, WI, USA). Point mutations were inserted using a site-direct mutagenesis technique to generate variations of the nucleoprotein sequence. The nucleocapsid protein sequence was amplified by PCR using forward (5′-TAATACGACTCACTATAGGGATGGCATCCCCTGCTGCACC-3′) and reverse (5′-CTAATCAGTGTTAACATCAA-3′) primers with PrimeSTAR Max DNA polymerase (Takara-Bio, Shiga, Japan). The amplicons were gel-purified and were used as templates for RNA transcription using a MEGAscript T7 Transcription Kit (Life Technologies, Carlsbad, CA, USA). The transcribed RNA was quantified using the OD value, the copies number was calculated, and the RNA was diluted with ribonuclease-free water containing 10 μg/mL of yeast RNA.

### Real-time RT-PCR assays

Real-time RT-PCR assays using upE and ORF1a sets [14,15] were also performed for virus detection using a QuantiTect Probe RT-PCR kit (QIAGEN) and LightCycler 480 Instrument (Roche, Basel, Switzerland) following the manufacturers’ protocols. The amplification conditions followed Corman *et al*. [14,15].

### Virus preparation for spiked samples

For sensitivity assays, Vero cells were infected with MERS-CoV, and incubated for 4 days. Cell supernatants were then collected and centrifuged at 1,500 × g for 30 min at 4°C, and the supernatants were treated with RNaseA (Nippongene, Tokyo Japan) at a concentration of 10 μg/mL for 30 min at 37°C to exclude miscellaneous RNA other than viral RNA.

### Pilot experiment

MERS-CoV RNA was obtained from pre-titrated viral stocks diluted with medium containing pharyngeal swabs obtained from healthy adults using the Universal Viral Transport for Viruses, Chlamydiae, Mycoplasmas and Ureaplasmas (Becton Dickinson and Company, Sparks, MD, USA). Viral isolation was also performed on Vero and Vero/TMPRSS2 cells constitutively expressing type II transmembrane serine protease (TMPRSS2) [30], which enhances cell entry and fusion formation of MERS-CoV [31,32]. Diluted viruses were inoculated on Vero and Vero/TMPRSS2 cells, and incubated for 60 min. Cells were then washed with PBS, and incubated in Dulbecco’s modified Eagle’s medium supplemented with 5% fetal calf serum at 37°C. The cytopathic effect was evaluated 5 days after inoculation. Clinical specimens (nasopharyngeal swabs) diagnosed as other respiratory pathogens by RT-PCR assays were used as negative controls. Informed, written consent was obtained at the time of sample collection from all patients. For the specimen diagnosed as human bocavirus, a LAMP assay was performed without the RT reaction.

## Results

### RT-LAMP primer design

The primer sets used in this study are listed in Table 1. RT-LAMP requires at least six specific sequences (F1, F2, F3, B3, B2, and B1), targeted by a minimum of four distinct primer sets. Two loop primers (LF and LB) are used to enhance amplification [22]; these primers target the regions between F1 & F2, and B1 & B2 regions, respectively. Generally, the reaction of RT-LAMP is performed for 1 h. However, the primers described here tend to generate products of self-construction because these were constructed to enhance the sensitivity of amplification. Therefore, the reaction was performed within 30 min to exclude non-specific reactions. It was confirmed that non-specific amplification did not take place within 30 min using negative control samples (data not shown).

**Table 1:** The primer set for MERS-CoV RT-LAMP assay

**Primers**	**Position (EMC, JX869059.2)**	**Sequence (5′ – 3′)**	Number of matched MERS sequences*
F3	28848–28866	GCTCCCAGGTGGTACTTCT	86/88
B3	29061–29042	cagtcccctcaatgtggaag	88/88
FIP (F1c + F2)	28939–28918	tcatggacccaaacgatgccatACTGGAACTGGACCCGAAG	77/88
	+ 28872–28890		88/88
BIP (B1c + B2)	28956–28977	GCTCCTTCAACTTTTGGGACGCtagtaccgggcgcgaatt	87/88
	+ 29028–29011		83-88
LF	28906–28891	cggaatgggagtgctg	88/88
LB	28978–29000	GGAACCCTAACAATGATTCAGCT	85/88

Capital letters indicate the sense strand; lowercase letters indicate the antisense strand.

*Eighty-one sequences were obtained from GenBank (JX869059, KJ829365, KJ813439, KJ713299, KJ713298, KJ713297, KJ713296, KJ713295, KJ650297, KJ650296, KJ650295, KJ650098, KJ556336, KJ477102, KJ156953, KJ156952, KJ156949, KJ156944, KJ156942, KJ156939, KJ156934, KJ156932, KJ156927, KJ156917, KJ156916, KJ156913, KJ156911, KJ156910, KJ156909, KJ156907, KJ156906, KJ156905, KJ156902, KJ156901, KJ156883, KJ156881, KJ156876, KJ156874, KJ156873, KJ156872, KJ156871, KJ156869, KJ156866, KJ156865, KJ156862, KJ156861, KF961222, KF961221, KF958702, KF917527, KF811035, KF745068, KF600652, KF600651, KF600647, KF600645, KF600644, KF600643, KF600639, KF600636, KF600635, KF600634, KF600632, KF600630, KF600628, KF600627, KF600623, KF600621, KF600620, KF600613, KF600612, KF192507, KF186567, KF186566, KF186565, KF186564, KC875821, KC776174, KC667074, KC164505, and KJ782550) and seven sequences were obtained online (England2-HPA, http://www.hpa.org.uk/webc/HPAwebFile/HPAweb_C/1317138176202: Jeddah_2014_C7149, C7569, C7770, C8826, C9055, and C9355, http://www.virology-bonn.de/index.php?id=46).

### Sensitivity and specificity of the RT-LAMP assay

**Figure 1:**
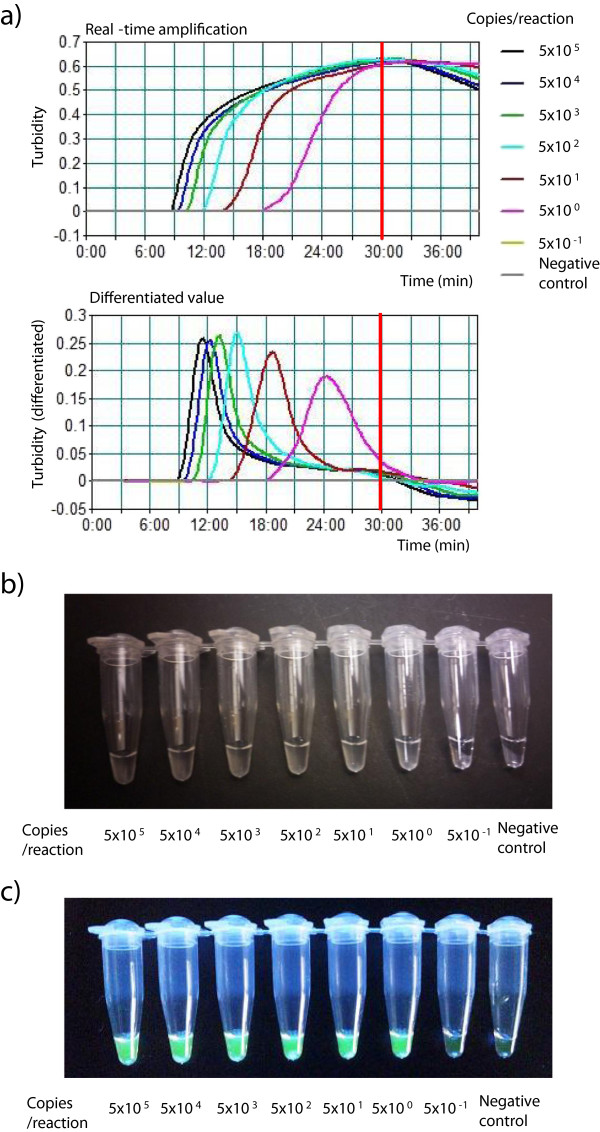
**Sensitivity of the MERS-CoV RT-LAMP assay. a)** Real-time amplification of MERS-CoV by RT-LAMP. Amplification of serially diluted MERS-CoV RNA was measured in real-time using a Loopamp real-time turbidimeter (LA-320C). The differentiated value at each dilution was calculated automatically, with values > 0.1 within 30 min (red line) considered positive. **b, c)** Detection of RT-LAMP amplicon by **b)** precipitation of magnesium pyrophosphate and **c)** fluorescence under ultra violet light. For fluorescence detection, 1-μL Fluorescence Detection Reagent was added to each reaction mixture. Fluorescent signals were detected under ultraviolet following completion of the amplification reaction. Successful amplification could be detected as green fluorescent light.

The detection limit of the RT-LAMP assay was determined using serially diluted MERS-CoV, and compared with those of real-time RT-PCR using upE and ORF1a assays [14,15] (Table 2). upE and ORF1a assays were able to detect 1.6 to 3.4 copies of MERS-CoV RNA, consistent with previous reports. The threshold for RT-LAMP within 30 min was also 3.4 copies, indicating a sensitivity equivalent to that of real-time RT-PCR [14,15].RT-LAMP amplification can be monitored in three DNA contamination-free manners. First is a real-time method that monitors the turbidity of the pyrophosphate precipitation using a turbidimeter (LA-320C) (Figure 1a). The differentiated value of each signal is calculated automatically at the same time during amplification, with values > 0.1 considered positive. RT-LAMP can also detect MERS-CoV without any specific instruments by detecting visible signals at the same level as real-time monitoring. Amplification can be determined through visual detection of magnesium pyrophosphate precipitation following completion of the reaction (Figure 1b). Furthermore, amplicons can be detected by means of green fluorescence under ultraviolet light by adding a fluorescence detection reagent to the mixture before the start of amplification (Figure 1c).

**Table 2:** Sensitivity of the RT-LAMP assay

**Copies/reaction**	**500,000**	**50,000**	**5000**	**500**	**50**	**5**	**0.5**	**Negative control**	**Sensitivity (copies)**
Real-time RT-PCR*									
upE	21.2	24.4	27.9	31.3	34.2	36.6	> 40	> 40	1.6
ORF1a	21.6	24.4	27.6	30.4	32	31.8	> 40	> 40	3.4
RT-LAMP**	11.33	12.13	13.07	15.44	21.44	22.33	> 30	> 30	3.4

*Threshold cycle.

**Time (min. s).

In addition to MERS-CoV, specific amplification via RT-LAMP was also tested using various respiratory viruses (Table 3). RT-LAMP was performed using pre-titrated viral stocks, as well as clinical specimens previously validated by PCR. MERS-CoV RT-LAMP was specific for MERS-CoV; other respiratory viruses, such as HCoV, SARS-CoV, RSV, influenza, PIV, ADV, and HMPV could not be amplified using the MERS-CoV primers.

**Table 3:** Specificity of the RT-LAMP assay

**Virus**	**Titer/reaction**	**Results (min)**
Coronaviruses		
MERS-CoV (EMC)	4 × 10^1^ TCID_50_	14.24
HCoV 229E (VR-740)	3 × 10^3^ PFU	> 30
HCoV 229E (Sendai-H/1121/04)	5 × 10^3^ PFU	> 30
HCoV NL63	2.5 × 10^2^ FFU	> 30
HCoV OC43 (VR-1558)	1.3 × 10^3^ TCID_50_	> 30
SARS-CoV (Frankfurt)	1 × 10^5^ PFU	> 30
Other Respiratory Viruses		
RSV A (Long)	1 × 10^1^ PFU	> 30
RSV A (A2)	1 × 10^2^ PFU	> 30
RSV B (18537)	1 × 10^2^ PFU	> 30
RSV B (WV/14617/85)	—*	> 30
HMPV (Sendai-H/2404/2003)	—*	> 30
PIV 1 (C-35)	3 × 10^4^ PFU	> 30
PIV 3 (C-243)	5 × 10^3^ PFU	> 30
ADV 3 (G.B.)	2.5 × 10^2^ TCID_50_	> 30
ADV 4 (RI-67)	1 × 10^2^ TCID_50_	> 30
ADV 7 (Gomen)	2.5 × 10^2^ TCID_50_	> 30
Flu A/California/7/2009 (H1N1pdm)	8 × 10^3^ TCID_50_	> 30
Flu A/Victoria/210/2009 (H3N2)	2.5 × 10^6^ TCID_50_	> 30
Flu B/Brisbane/60/2008	2.5 × 10^4^ TCID_50_	> 30

*Titer unknown, but was confirmed by PCR.

PFU: plaque forming unit.

FFU: focus forming unit.

TCID50: 50% tissue culture infectious dose.

### Pilot experiments

As MERS-CoV clinical isolates are not available in Japan, a pilot study was performed using MERS-CoV laboratory isolates diluted with medium containing pharyngeal swabs obtained from healthy adults. Viral detection was carried out using both RT-LAMP and real-time RT-PCR assays, and with virus isolation using Vero and Vero/TMPRSSS2 cells (Table 4). Viral isolation from Vero cells required at least 500 copies of MERS-CoV, followed by incubation at 37°C for 5 days. It contrast, although it has been reported that TMPRSS2 enhances the entry and fusion formation of MERS-CoV [31,32], Vero/TMPRSS2 cells exhibited syncytium formation with 23.2 copies of MERS-CoV within 2 days of incubation.

**Table 4:** Detection of MERS-CoV diluted with medium containing pharyngeal swabs

**Copies/50 μL**	**500,000**	**50,000**	**5000**	**500**	**50**	**5**	**0.5**	**Negative control**	**Sensitivity (copies)**	**Time required**
Virus isolation*										
Vero	6/6	6/6	6/6	3/6	0/6	0/6	0/6	0/6	500	5 d
Vero/TMPRSS2	6/6	6/6	6/6	6/6	5/6	0/6	0/6	0/6	23.2	2 d
**Copies/reaction**	**50,000**	**5000**	**500**	**50**	**5**	**0.5**	**0.05**			
Real-time RT-PCR**										
upE	22.0	25.3	28.7	32.3	33.2	> 40	> 40	> 40	1.6	2 hr
ORF1a	21.9	25.2	28.5	32.2	32.5	> 40	> 40	> 40	1.6	2 hr
RT-LAMP***	11.00	11.16	12.18	13.48	18.04	23.12	> 30	> 30	0.7	30 min

*Positive number/tested number.

**Threshold cycle.

***Time (min. s).

The real-time RT-PCR was highly sensitive for MERS-CoV, with upE and ORF1a assays capable of detecting as few as 1.6 copies of MERS-CoV RNA. The RT-LAMP was also able to detect viral RNA at levels as low as 0.7 copies, showing equivalence with the RT-PCR assay. Viral isolation using Vero/TMPRSS2 cells was more sensitive than that of Vero cells; however, genetic diagnostic assays were consistently more sensitive than culture-based methods. These data suggest that the RT-LAMP assay is capable of detecting MERS-CoV with a sensitivity similar to that of real-time-RT-PCR, even in clinical specimens.

Next, clinical specimens previously diagnosed as other respiratory pathogens were tested using the RT-LAMP assay (Table 5). All reactions were negative, with no cross reactivity for other respiratory viruses, indicating a high degree of specificity for the RT-LAMP assay. Collectively, these results suggest that the MERS-CoV RT-LAMP assay is useful for epidemiological surveillance in suspected clinical cases.

**Table 5:** Detection of MERS-CoV using clinical specimens diagnosed as other respiratory viral infections

**Diagnosed pathogen**	**Results (min)**
Positive control	
*MERS-CoV (10^4^ copies)	9.00
Coronaviruses	
HCoV OC43	> 30
HCoV NL63	> 30
HCoV HKU1	> 30
Other Respiratory viruses	
RSV A	> 30
RSV B	> 30
HMPV	> 30
PIV 1	> 30
PIV 2	> 30
PIV 3	> 30
PIV 4	> 30
Rhinovirus	> 30
Bocavirus	> 30
Flu A H1 (Russian)	> 30
Flu A H1 (2009 pdm)	> 30
Flu A H3	> 30
Flu B (Yamagata)	> 30
Flu B (Victoria)	> 30
Flu C	> 30
Measles virus	> 30
Rubella virus	> 30

*Synthesized RNA obtained from N protein region.

### RT-LAMP validation for mismatched sequences

**Figure 2:**
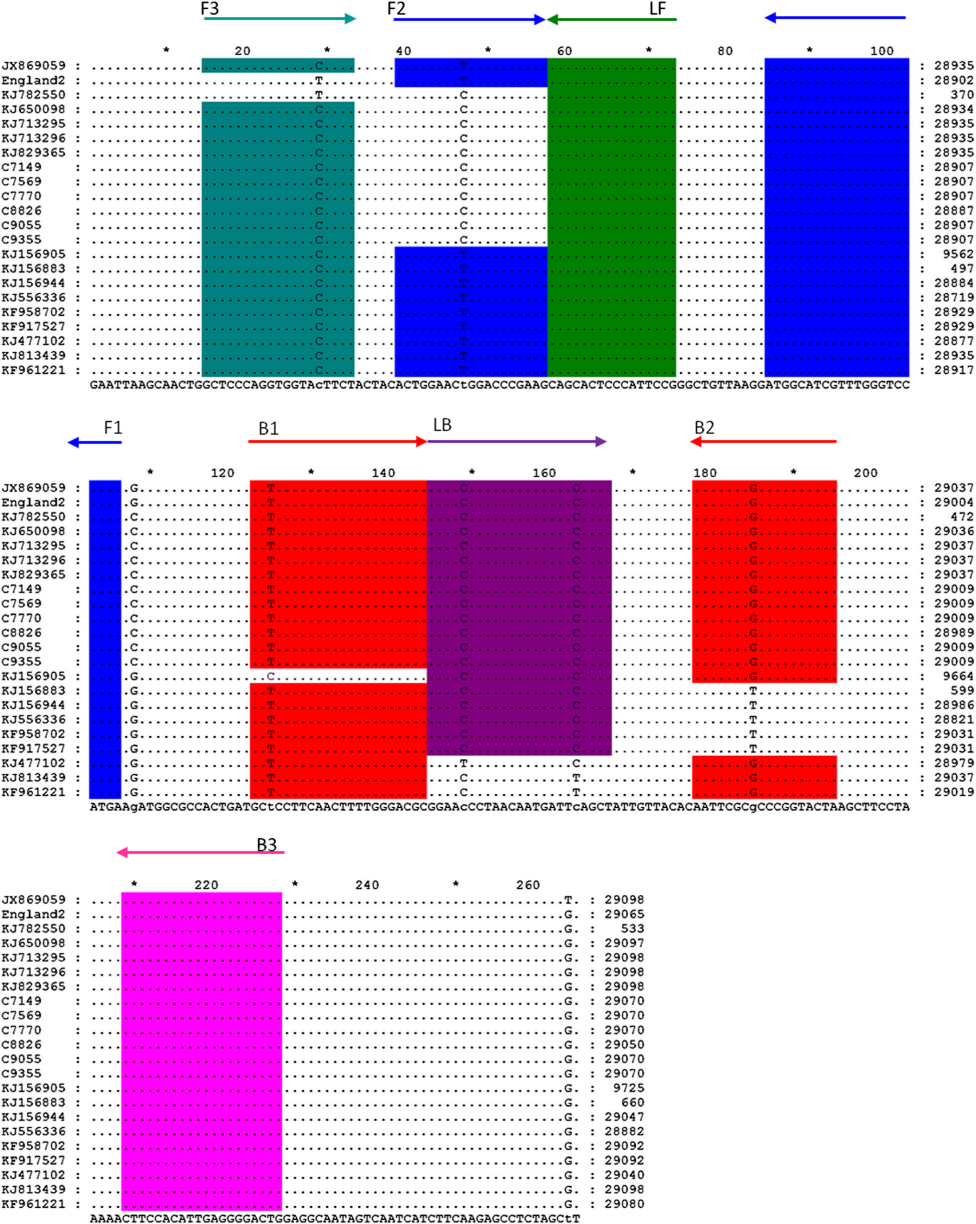
**Nucleotide mismatches in the MERS-CoV sequences.** The MERS-CoV sequences that have mismatches with the RT-LAMP primer sets were identified in an alignment based on the sequence of the EMC isolate (JX869059.2). The positions of six essential regions (F3, F2, F1, B3, B2, and B1) and loop primers (LF and LB) are indicated. The accession numbers of the MERS-CoV sequences used in the alignment were as follows: JX869059, KJ782550, KJ650098, KJ713295, KJ713296, KJ829365, KJ156905, KJ156883, KJ156944, KJ556336, KF958702, KF917527, KJ477102, KJ813439 and KF961221. Seven MERS-CoV sequences available online (England2, Jeddah_2014_C7149, C7569, C7770, C8826, C9055 and C9355) were also used. The alignment was performed using GeneDoc ver. 2.7 (http://www.nrbsc.org/gfx/genedoc/).

The primer set was constructed based on the conserved region of the nucleocapsid protein sequence of the EMC isolate of MERS-CoV (GenBank accession no JX869059.2). The number of sequences deposited in GenBank is increasing, and this region of MERS-CoV exhibits several sequences variation. Therefore, the primer sequences were checked against the available MERS-CoV sequences. Eighty-one MERS-CoV nucleocapsid sequences were collected from GenBank, along with seven other sequences reported online (England2-HPA, Jeddah_2014_C7149, C7569, C7770, C8826, C9055, and C9355]. An alignment was constructed using the EMC isolate and mismatched sequences (Figure 2). The RT-LAMP primers matched most of the 88 sequences. Primers B3 and LF matched them completely. Primer F3 had one nucleotide mismatch for two sequences. Primer LB had one nucleotide mismatch for one sequence and had another mismatch for two sequences. Primer BIP had one nucleotide mismatch in B1 region of one sequence and had another mismatch in the B2 region of five sequences. Primer FIP had a one-nucleotide mismatch in the F1 region of 11 sequences (Table 1). The efficiency of RT-LAMP for these mismatched sequences was evaluated using an RNA template transcribed from a 1242-bp of PCR amplicon from the nucleocapsid gene sequence of MERS-CoV (Table 6). The sensitivity of RT-LAMP using the RNA control template of the EMC isolate was 15.8 copies and tended to be lower than that using viral RNA as the template. This might be caused by a difference in RT efficiency or the secondary structure of RNA due to the difference in length. The mismatches in the F3, F2, B1, and LB region did not markedly affect the amplification efficiency, and the sensitivity was equal to or twice that of the EMC isolate. The mismatch in the B2 region affected amplification slightly, and showed a fivefold decrease in the sensitivity (73.4 copies). This mismatch in B2 was seen in 5 sequences (KJ156883, KJ156944, KF556336, KF958702, and KF917527) of the 88 MERS-CoV sequences checked in this study. Nevertheless, these results suggest that the RT-LAMP primer set facilitates amplification of the remaining 83 sequences to the same level as the EMC isolate.

**Table 6:** Evaluation of RT-LAMP amplification using mismatched sequences

**Accession**	**Name**				**Sensitivity (copies)**
JX869059	EMC				15.8
**Representative sequence**		Nucleotide position^*^	**Substitution**	**Region in primer**	**Sensitivity (copies)**
	England2	28862	C to T	F3	7.3
KJ782550	Greece-Saudi Arabia_2014	28862	C to T	F3	15.8
		28880	T to C	FIP (F2)	
KJ650098	Camel/Qatar_2_2014	28880	T to C	FIP (F2)	34.1
KJ156905	Riyadh_7b_2013	28958	T to C	BIP (B1)	15.8
KF917527	Jeddah-Camel-1	29018	G to T	BIP (B2)	73.4
KJ477102	NRCE-HKU205	28982	C to T	LB	39.7
KF961221	Qatar3	28996	C to T	LB	7.3

*Based on EMC isolate (JX869059.2).

## Discussion

As described above, definitive MERS-CoV diagnosis requires amplification of at least two different virus-specific genomic targets according to the case definition reported on 3 July 2013 by the WHO. However, only two real-time RT-PCR targets, upE and ORF1a, are currently available for MERS-CoV detection with high specificity and sensitivity [14,15]. Indeed, two previous cases reported by the Italian government had to be reclassified as probable MERS-CoV infections, as they were unable to fulfill these criteria (WHO, GAR, MERS-CoV summary and literature update – as of 20 September 2013, http://www.who.int/csr/disease/coronavirus_infections/update_20130920/en/index.html). Additional sensitive and specific genetic diagnostic methods are therefore needed to provide reliable MERS-CoV diagnoses.

This study describes a novel genetic diagnostic method for MERS-CoV based on the RT-LAMP assay, with a sensitivity and specificity equal to that of the upE and ORF1a RT-PCR assays. This assay was also able to detect MERS-CoV RNA in experimentally obtained nasopharyngeal swabs, and never showed cross reactivity to other respiratory viruses, even in the case of clinical specimens.

The RT-LAMP method requires only a single temperature for amplification, with results usually available in less than 1 h by observing magnesium pyrophosphate precipitate or fluorescence signals by the naked eye [21,22]. Although RT-LAMP amplification can be monitored in real-time using a turbidimeter [23], the assay can also be performed using basic laboratory equipment, such as a heat block and water bath. The method has been validated using various respiratory viruses, as well as more diverse pathogens, such as bacteria [33-35], protozoa [36-38], and parasites [39,40]. Furthermore, the reagents necessary to perform RT-LAMP are commercially available. Recently, Abd El Wahed et al., reported a genetic diagnostic method for MERS-CoV based on reverse transcription isothermal recombinase polymerase amplification (RT-PPA) assay. The method is highly sensitive and can detect 10 copies of virus RNA within 3 to 7 minutes. Therefore, it is useful for the diagnosis of MERS-CoV as well. However, RT-RPA requires a specific tubescanner for detection. By contrast, the RT-LAMP assay enables detection of amplicons by observing a magnesium pyrophosphate precipitate or fluorescence signals with the naked eye with no requirement for any specialized instruments. Taken together, the specificity and sensitivity of the RT-LAMP assay described here, in combination with its accessibility and ease of use, make this assay a valuable tool for the diagnosis and epidemiologic surveillance of human MERS-CoV infection, especially for field use.

The primer sets described in this study matched to most of the available sequences. However, several sequences had mismatches with the primers. The effect of these mismatches on the RT-LAMP efficiency was evaluated using a synthesized control RNA template: most of the mismatches did not affect the amplification efficiency of RT-LAMP. However, the substitution in B2 region of the primer, namely, a G to T substitution at position 29018 in the nucleocapsid gene sequence of the EMC isolate, was present in five MERS-CoV sequences and caused a slight decrease in sensitivity. Of the five sequences, KJ156883 (Asir_1_2013) belongs to the Buraidah_1 clade, and is the only sequence in this clade with such a substitution [41]. The other sequences (KJ156944, Riyadh_5_2013; KJ556336, Jeddah_1_2013; KF958702, Jeddah_human1; KF917527, Jeddah_Camel1) belong to the Riyadh_3 clade [42,43]; none of the other sequences in this clade have a substitution according to the alignment analysis. Therefore, the substitution does not represent a major population in this clade. Although it is important to improve the primers to increase their sensitivity for sequences in the Riyadh_3 clade by using mixed bases, the results of this study suggest that the RT-LAMP primer is useful for detecting the majority of the prevalent MERS-CoV strains.

The success rate of MERS-CoV detection is dependent on the collection of clinical specimens. Drosten *et al*., reported that up to 10^6^ copies/mL of MERS-CoV RNA are present in lower respiratory tract specimens, such as tracheobronchial secretions, and bronchoalveolar lavage. In contrast, only small amounts of viral RNAs were detected in upper respiratory tract specimens and other tissue [44]. The RT-LAMP primers described here were designed based upon the nucleocapsid protein sequence of MERS-CoV, due to the unique coronaviral replication system. Although coronaviruses generate subgenomic mRNAs to produce each viral protein, all subgenomic mRNAs contain nucleoprotein sequences, as they are located on the 3′end of the coronavirus genome [45-47]. This implies that the RT-LAMP procedure described here will exhibit a high degree of sensitivity for specimens containing cellular components.

## Conclusions

This study developed a RT-LAMP assay for the MERS-CoV, which was capable of detecting as few as 3.4 copies of MERS-CoV RNA, and was highly specific, with no cross-reaction with other respiratory viruses. These results suggest that the RT-LAMP assay described here is a useful tool for the diagnosis and epidemiologic surveillance of human MERS-CoV infections.

## Abbreviations

CoV: Coronavirus; FFU: Focus forming unit; MERS: Middle East respiratory syndrome; ORF: Open reading frame; PBS: Phosphate-buffered saline; PFU: Plaque forming unit; RT-LAMP: Reverse transcription-loop-mediated isothermal amplification; TCID50: 50% tissue culture infectious dose; TMPRSS2: Transmembrane protease, serine 2; upE: Upstream E.

## Competing interests

The authors declare that they have no competing interests.

## Authors’ contributions

All authors participating in the planning of the project. SS and TsuN constructed the RT-LAMP primer set and evaluated the sensitivity. TY, SA, TK, and TaN participated in the pilot experiments. KS participated in all experiments and wrote manuscript. SM is the leader of the project. All authors read and approved the final manuscript.
